# 

**Published:** 1922-06

**Authors:** 


### RECENT APPOINTMENTS.

### Public Health.

Dr. H. M. Melville Rees, Medical Superintendent of Walton Sanatorium, Chesterfield, to be Tuberculosis Officer for Pembrokeshire.

Mr. William J. Cox, M.B., Ch.B., D.P.H., late Medical Officer of Health, Borough of Chelmsford, to be Medical Officer of Health, Urban District of Watford.

Dr. J. A. Harbison, M.B., B.Ch., D.P.H., late Deputy M.O.H. and T.O., Barrow-in-Furness, to be Asst. M.O.H. and T.O. for the Metropolitan Borough of Deptford.

Mr. John Charles Saunders, M.B., B.Ch., B.A.O., D.P.H., formerly Assistant R.M.O., Peamount Sanatorium, Co. Dublin, R.M.O., Eccleston Hall Sanatorium, St. Helen's, &c., to be Tuberculosis Officer and Resident M.O. at Hefferston Sanatorium, County Borough of Warrington.

Mr. Harry Mainwaring Holt, formerly Reserve M.O., Leeds Public Dispensary; House Physician and Reserve Obst. Officer, Leeds General Infirmary; and Reserve M.O., Ministry of Pensions Orthopædic Hospital, Oxford, &c., to be Additional Assistant Medical Officer for Maternity and Child Welfare for the City of Leeds.

Dr. Harry Osborne, M.R.C.S., L.R.C.P., M.D., D.P.H., formerly House Physician, Manchester Royal Infirmary; House Surgeon, Manchester Southern Hospital; M.O. at Children's Hospital, Gartside Street, Manchester; Assistant Bacteriologist, Manchester Public Health Laboratory, &c., to be Medical Officer of Health for the County Borough of Salford. He has been in the service of the Salford Public Health Department for 14 years, and when the new appointment was made was Deputy Medical Officer.

---

Mr. T. Feilding Johnson has been re-elected chairman, and Col. Bond has resigned the vice-chairmanship, of the Leicester Royal Infirmary.

### PARLIAMENTARY QUESTIONS AND ANSWERS.

April 26.—Sir A. Mond informed Mr. T. Thomson that the number of completed houses in State-aided schemes was 130,335, and that 52,417 were in course of construction. A further 21,151 had been authorised but not yet begun.

May 3.—Sir A. Mond, in reply to Mr. C. Edwards, stated that it was laid down by the Government as one of the fundamental conditions for a grant from the Voluntary Hospitals Commission that assistance should ordinarily be based on the amount of new money raised or in sight, and the Commission had no power to vary this rule.

Sir A. Mond informed Mr. Doyle that he was hoping to introduce legislation with a view to effecting a substantial improvement in the milk supply of the country.

May 4.—Sir A. Mond informed Sir J. D. Rees that, so far as he was aware, only an extremely small proportion of the local authorities concerned had adopted the immediate self-disinfection policy of the Society for the Prevention of Venereal Disease.

Sir A. Mond informed Mr. T. Griffiths that the admission of students to university institutions was essentially for the discretion of the university authorities, with whose autonomy the Government desired to interfere as little as possible. The Government had no information indicating that the facilities for university education in medicine for women were inadequate.

May 10.—In reply to Mr. Myers, Sir A. Mond stated that the Rockefeller Foundation had made a gift of 2,000,000 dollars for providing and equipping a school of hygiene, and had recently acquired an admirable site for the purpose in Bloomsbury. A Government grant will be applied to staffing and maintaining the school, the object of which is to promote hygiene and health by providing facilities for the medical practitioner, and for research, in preventive medicine.

May 11.—Mr. Macpherson informed Lieut.-Col. Spender Clay that 52 hospitals were maintained by the Ministry of Pensions, at which approximately 10,000 officers, nurses and men were receiving in-patient treatment.

May 15.—Sir A. Mond informed Lieut.-Col. Hurst that the Registrar-General had under consideration, with a view to amendment, the regulation which at present debars applications for registering births on behalf of children over seven years of age.

### NEW BILLS AND LEGISLATIVE PROPOSALS.

May 10.—Lieut.-Commander Kenworthy moved for leave to introduce a Bill "to prevent the application of public money to vivisection experiments." Dr. Murray opposed and leave was refused by 170 to 102.

May 11.—A Bill was presented by Mr. Rendall "to amend the law relating to the marriage of persons with their nephew or niece by marriage."

On the motion for the second reading of the Bill for confirming Provisional Orders made by the Minister of Health extending the boundaries of the county boroughs of Leeds and Bradford, an animated debate took place, and the rejection of the Bill was ultimately carried by 109 to 35.

The Local Government and other Officers Superannuation Bill passed through Committee and was reported to the House.

May 15.—The Bread Bill introduced by Mr. Inskip was read a second time. This Bill legalises the use of self-raising flour in the making of bread.

### THE COLLEGE OF NURSING.

The annual meeting and conference of the College of Nursing will be held in London on Thursday and Friday, June 22 and 23, when the laying of the foundation stone of the College of Nursing building and the official opening of the Cowdray Club at 20 Cavendish Square will also take place.

Nurses and other professional women are eligible for membership of this club. The annual subscriptions will be £2 10s. town, £1 10s. country; members of the College of Nursing, half price. For the first 2,000 members joining, no entrance fee will be charged. About £100,000 has been spent in acquiring, adapting and furnishing the beautiful house. Bedrooms will be available at an inclusive charge of 5s. a night; the catering and cooking will be of a high standard, but very moderate charges will be made for meals.

Miss Evelyn Leggatt, R.R.C., who was trained at King's College Hospital under the late Miss Katharine Monk, has been appointed Club Superintendent. She nursed in South Africa during the South African War and in Serbia during the war with Bulgaria. In 1914 she was called up on the Q.A.I.M.N.S. Reserve, and was stationed for one year at Woolwich, in charge of the Sick Officers' Mess. She was Matron of the Vincent Square Hospital for Sick Nurses from 1915 until demobilisation in 1919. Miss Leggatt has travelled in many countries, and holds, among other certificates, the Cordon Bleu of the National Training School of Cookery.

Miss E. M. Litten, for many years private secretary to the late Sir Henry Burdett, has been appointed Club Secretary. She has expert knowledge of secretarial duties, committee work, finance and business routine; she was associated with "The Hospital" and "The Nursing Mirror," and was also sub-editor of "Burdett's Hospitals and Charities." She is familiar with press matters and publishing, and her long association with nurses will be invaluable in her work.

### BOYS AND GIRLS IN BORSTAL INSTITUTIONS.

The attention of the State Children's Association having been drawn to the punishments in the Borstal Institution for Girls at Aylesbury, recorded in the Prison Commissioners' Report for 1920-21, the Executive Committee addressed a letter to the Commissioners pointing out that much general anxiety was felt as to the punishments of solitary confinement and the use of handcuffs, which appeared to be greatly in excess of the numbers for 1918.

In their reply the Prison Commissioners explained that a number of the girls sent to Aylesbury during 1920-21 had been leading wild and dissolute lives under war conditions, and their treatment presented extraordinary difficulties. The punishments in question had already been the subject of a special inquiry. Of the 33 cases in which the body belt with swivel handcuffs had been employed, 23 concerned eight girls who were mentally deficient and were removed to a special institution at the earliest opportunity. The other ten cases were of ten girls who were influenced by the atmosphere of violence which spread in the Institution owing to the presence of the mental deficients. With regard to the 74 cases of confinement to cells, an attempt was being made to do without the punishment of confining to rooms altogether, and to rely entirely on the prospect of loss of privileges and reduction in grade for maintaining the standard of conduct. The number of restraints at Aylesbury from the end of March, 1921, to January 27, 1922, was three only, and those of confinement to cells 15, as compared with the 33 and 74 recorded above.

To the Association's inquiry whether an expert examination could not be made into the mental condition of boys or girls committed to Borstals before their reception into those institutions, the Commissioners replied that they had issued a circular impressing on all Medical Officers of Prisons the necessity of making very careful reports to the Courts of Justice on all young offenders of Borstal age committed for trial or remanded for inquiries. No such persons should, in their opinion, be recommended for Borstal training as are incapable of mental progress and of receiving trade instruction. They have instituted reception classes at Feltham for boys, and at Aylesbury for girls. All inmates are placed in one of these classes for such time as will enable their conduct to be observed and their mental condition to be tested by modern scientific methods. Should an inmate be considered unfit for Borstal training, the case is specially considered with a view to early discharge, either on licence or on remission of sentence. The Commissioners endeavour to provide girls, equally with boys, with opportunities to learn industries, and with such occupations and recreations as will improve physique and stimulate mental activity.

### H.M. STATIONERY OFFICE PUBLICATIONS.

[The prices quoted include postage.]

Command Papers: (1633) Ministry of Health, Statement showing for each Borough and other Urban District for England and Wales and for 100 typical Rural Parishes the amount of the local rates per £ of assessment value (1920-1 and 1921-2), and the assessable values in force at the beginning of the year 1921-2. 1s. 1½d.
(1635) National Health Insurance Fund Accounts. Appendix. Approved Societies and Insurance Committees. Receipts and payments, 1919. 4d.
(1644). Ministry of Labour. Inter-departmental Committee on Health and Unemployment Insurance. First and second interim reports. 4d.

Miners' Nystagmus Committee, First Report of the Medical Research Council, Special Report No. 65. 1s. 8d.

Health Visitors, Conditions for the certification and registration of (Scottish Board of Health). 3d.

Roster of Medical Practitioners who may issue medical certificates for use by the Oversea Settlement Office and the Immigration Services of the Oversea Dominions. 10½d.

Royal Commission on London Government. Minutes of Evidence, Part III. 6s. 5d.

House of Commons Bills:
No. 49. Flour and Bread (Standard Quality). To regulate the composition of flour milled as standard flour, and of bread sold as standard bread. 3d.
No. 117. Local Government and Other Officers' Superannuation (as amended by Standing Committee A). 7d.

Circular 310. Public Health (Foreign Meat) Regulations. To Port Sanitary Authorities and certain Sanitary Authorities. England and Wales. May 5. 2d.

Fœtal Death, Report on the Causation of. 10s. 4½d.

A Graphical Cost Analysis of Cottage Building (Special Report No. 6 of the Building Research Board). 2s. 8½d.

### Statutory Rules and Orders.

National Health Insurance: Exempt Persons' Regulations, March 28, 1922. 2d.

Factory and Workshop. Dangerous and Unhealthy Industries. The India-rubber Regulations, March 31, 1922. 3d.

National Health Insurance. Termination of Sanatorium Benefit Amendment Regulations, May 1. 2d

### OFFICIAL REPORTS RECEIVED.

County Borough of Blackburn: Dr. W. Allen Daley (M.O.H. and S.M.O.) to the Education Committee, 1921.
City of Birmingham: Dr. G. A. Auden (S.M.O.) to the Education Committee, 1921.
Borough of Glossop: Dr. E. H. M. Milligan (M.O.H. and S.M.O.) to the Education Committee, 1921.
Glossop Urban Sanitary Authority: Report of the M.O.H. and of the Sanitary Inspector, 1921.
Glossop-Dale R.D.C.: Report of the M.O.H., 1921.
Borough of Cambridge: Report of the M.O.H. and S.M.O. and of the Borough Dentist, 1921.
County Borough of Blackburn: Report of the M.O.H., 1921.
Children's Fresh-Air Mission, 1921.
Central Committee for the Care of Cripples, 1921.
National League for Health, Maternity and Child Welfare 1921.

**Hospitals and Homes:—**

Royal Southern Hospital, Liverpool, 1921.
City of London Mental Hospital, Stone, near Dartford, 1921.
Minehead and District Hospital (Luttrell Memorial), 1921.
Harrogate Infirmary, 1921.
Skipton and District Hospital, 1921.
East-End Mothers' Lying-In Home, 394, 396 and 398 Commercial Road, E.1, 1921.
Royal London Ophthalmic Hospital, 1921.
Beckett Hospital and Dispensary, Barnsley, 1921.
Bristol General Hospital, 1921.
King Edward VII. Sanatorium, Midhurst. Year to July, 1921.

### MEMORANDA FOR THE MONTH.

A Fancy Fair in aid of the British Home and Hospital for Incurables is to take place on Wednesday, June 7, in the grounds of the Institution, Crown Lane, Streatham.

A pageant and bazaar are being held on May 30-31, at Bournemouth, in aid of the Royal Boscombe and West Hants Hospital.

The annual dinner of the Federation of Medical and Allied Services will be held at the Café Royal, Regent Street, W.1, on June 7, at 7.15 p.m. Sir Berkeley Moynihan will preside, and Sir Alfred Mond, Lord Islington and Mr. C. Addison, M.P., intend to be present. Members should make early application for tickets (10s. 6d. each) to the Medical Director, 12, Stratford Place, W.1.

Alexandra Day has been fixed for June 21, when it is hoped to raise £50,000 in London. Since it was founded in 1912, it has raised over one million pounds. As a result of last year's collection in London, hospitals received £25,165; nursing associations, £1,555; dispensaries, £745; convalescent homes, £1,620; infant welfare, £835; and Queen Alexandra's special charities, £2,995. All the roses are made by cripples, 320 of whom are kept working all the year round.

A course of advanced lectures on infant care for crèche nurses and probationers, by Drs. Naomi Tribe and Jane Hawthorne, began on May 18 at Carnegie House, 117 Piccadilly, W. 1. A lecture is given every Thursday, at 7.30 p.m., until July 20. Tickets (1s. 3d. for each lecture) and full particulars can be obtained from Miss Halford, Honorary Secretary, National Association for the Prevention of Infant Mortality, or from Miss Maddock, Secretary of the National Society of Day Nurseries, both at the above address. A course on home nursing and first aid in connection with mothers and children is also in progress on Mondays at 6 p.m. until July 17. Tickets (2s. 6d. for the course, 1s. for one lecture) can be obtained from Miss Reed, National League f r Health, Maternity and Child Welfare, at Carnegie House.

### THE INVALID CHILDREN'S AID ASSOCIATION.

At the annual meeting of the Invalid Children's Aid Association, Lord Burnham said that the society went to the very root of the social question in the cure and treatment of the sick poor. He believed our hospital system, in the freedom of its spirit, its tradition, and its initiative, to be far ahead of any other in the world. It elicited a degree of self-sacrifice and sympathy which was certainly not brought out by the State-controlled or municipal establishments which were the rule elsewhere. In one matter, however, he thought it failed badly. Whilst we offered to all classes of the people the finest skill and expert advice that can anywhere be found in order to heal their wounds and alleviate their suffering, we seemed to imagine that the moment a patient was discharged from the hospital we had made him whole and restored him to the efficiency of his normal life. He thought they would never have an order of things that was satisfactory either from the medical or the lay point of view unless they had regularly attached and subsidiary to the great hospitals a sufficient series of convalescent homes for recovery and recuperation. In respect of sick children, and especially for those afflicted with tubercular disease, that association came in and, in a sense, supplemented and perfected the work done in the hospitals. He hoped that when people of goodwill were thinking of the general appeal that was being made on behalf of the hospitals they would not forget the immediate necessities of that society, without which no children's hospital and no ward in a general hospital dealing with children could properly discharge their duty.

In 1921 34,000 cases were dealt with, and of the £60,306 raised by the parent society and branches, £42,448 was allocated specially to case work. This year a great effort is being made to render self-supporting the convalescent homes at Broadstairs, Worthing, Seaford, Dover, Berkhamsted and Stonebridge Park. To achieve this object £4,000 is needed, and an endeavour is being made to do so by a Million Penny Fund.

